# Hyaluronic acid coatings as a simple and efficient approach to improve MSC homing toward the site of inflammation

**DOI:** 10.1038/s41598-017-08687-3

**Published:** 2017-08-11

**Authors:** Bruna Corradetti, Francesca Taraballi, Jonathan O. Martinez, Silvia Minardi, Nupur Basu, Guillermo Bauza, Michael Evangelopoulos, Sebastian Powell, Claudia Corbo, Ennio Tasciotti

**Affiliations:** 1 Department of Nanomedicine, Houston Methodist Research Institute, Houston, TX 77030 USA; 2 Department of Life and Environmental Sciences, Università Politecnica delle Marche, 60131 Ancona, Italy; 3 Center for Biomimetic Medicine, Houston Methodist Research Institute, Houston, TX 77030 USA; 4 Centre for NanoHealth, Swansea University Medical School, Swansea University Bay, Singleton Park, SA2 8PP Wales, UK; 5 Department of Orthopaedic & Sports Medicine, The Houston Methodist Hospital, Houston, TX 77030 USA

## Abstract

A major challenge in regenerative medicine is to improve therapeutic cells’ delivery and targeting using an efficient and simple protocol. Mesenchymal stem cells (MSC) are currently employed for the treatment of inflammatory-based diseases, due to their powerful immunosoppressive potential. Here we report a simple and versatile method to transiently overexpress the hyaluronic acid (HA) receptor, CD44, on MSC membranes, to improve their homing potential towards an inflammatory site without affecting their behavior. The effect of HA-coatings on murine MSC was functionally determined both, *in vitro* and *in vivo* as a consequence of the transient CD44 overexpression induced by HA. Data obtained from the *in vitro* migration assay demonstrated a two-fold increase in the migratory potential of HA-treated MSC compared to untreated cells. In an LPS-induced inflamed ear murine model, HA-treated MSC demonstrated a significantly higher inflammatory targeting as observed at 72 hrs as compared to untreated cells. This increased accumulation for HA-treated MSC yielded a substantial reduction in inflammation as demonstrated by the decrease in the expression of pro-inflammatory markers and by the induction of a pro-regenerative environment.

## Introduction

Mesenchymal stem cells (MSC) are promising candidates for cell-based therapy to treat several diseases^1–5^. They are compelling to consider as vehicles for delivery of biologically active agents^1^. Although systemic administration of MSC is considered the optimal route for clinical trials^6^, the major challenge is to efficiently deliver them to a target location and enhance engraftment^7^. The limitation is mainly represented by the way MSC use to exert their therapeutic impact, through the secretion of trophic and immunomodulatory factors soon following injection^8^.

In fact, MSC exhibit minimal persistence following systemic administration with low efficiency in targeting diseased or inflamed tissues^9–11^. The mechanism governing the MSC potential to home to sites of inflammation/injury is mediated by key ligands, including adhesion ligands (i.e., E-selectin glycoprotein ligand-1), or homing ligands that bind to intercellular adhesion molecules or chemokine receptors^12, 13^. However, the expression of these ligands is not consistent or are poorly expressed in MSC^14^. In addition, the expression of these ligands can be impacted by culture conditions effectively limiting their potential when long *in vitro* expansion is required for cell-therapy applications. For this reason, so far several studies have been proposed aiming at transiently enhancing MSC homing toward the site of inflammation following systemic administration^15^. They both exploit MSC genetic^8, 16, 17^ or enzymatic modifications^18^. MSC alterations through the use of retrovirus vectors encoding homing receptors (i.e., C-X-C motif chemokine receptor 4 or the a4 subunit of the very late antigen-4-integrin) have been developed^19, 20^. Other groups have achieved enhanced homing efficiency to specific tissues by functionalizing the cell surface through conjugation of specific antibodies or proteins^21–23^. To this extents, the active site of P-selectin glycoprotein ligand-1 found on leukocytes was immobilized on MSC surface through a biotin–streptavidin bridge enhancing a rolling response under shear stress conditions^18^. In contrast to enzymatic and covalent modifications, Sarkar *et al*. recently described an alternative cell membrane engineering approach to immobilize the ligand of interest through coalescence of ligand-carrying lipid vesicles into the cell membrane for a short duration^24^.

The hyaluronic acid (HA) is a large glycosaminoglycan, which serves as one of the important components of the extracellular matrix. Numerous functions have been ascribed to it, including immune activation, promotion of proliferation, migration, and intracellular signaling^25, 26^. Its role as a mediator of MSC homing is particularly interesting as its production increases during inflammation and injury^27^. To date, researchers demonstrated that when its receptor (CD44) is blocked using anti-CD44 antibodies, intravenous administration of MSC into a kidney mice model resulted in a reduced renal healing compared to controls. Enzymatic modifications have also been applied to engineer CD44 on the MSC membrane resulting in an improved migration compared to unmodified cells^28^.

**Figure 1:**
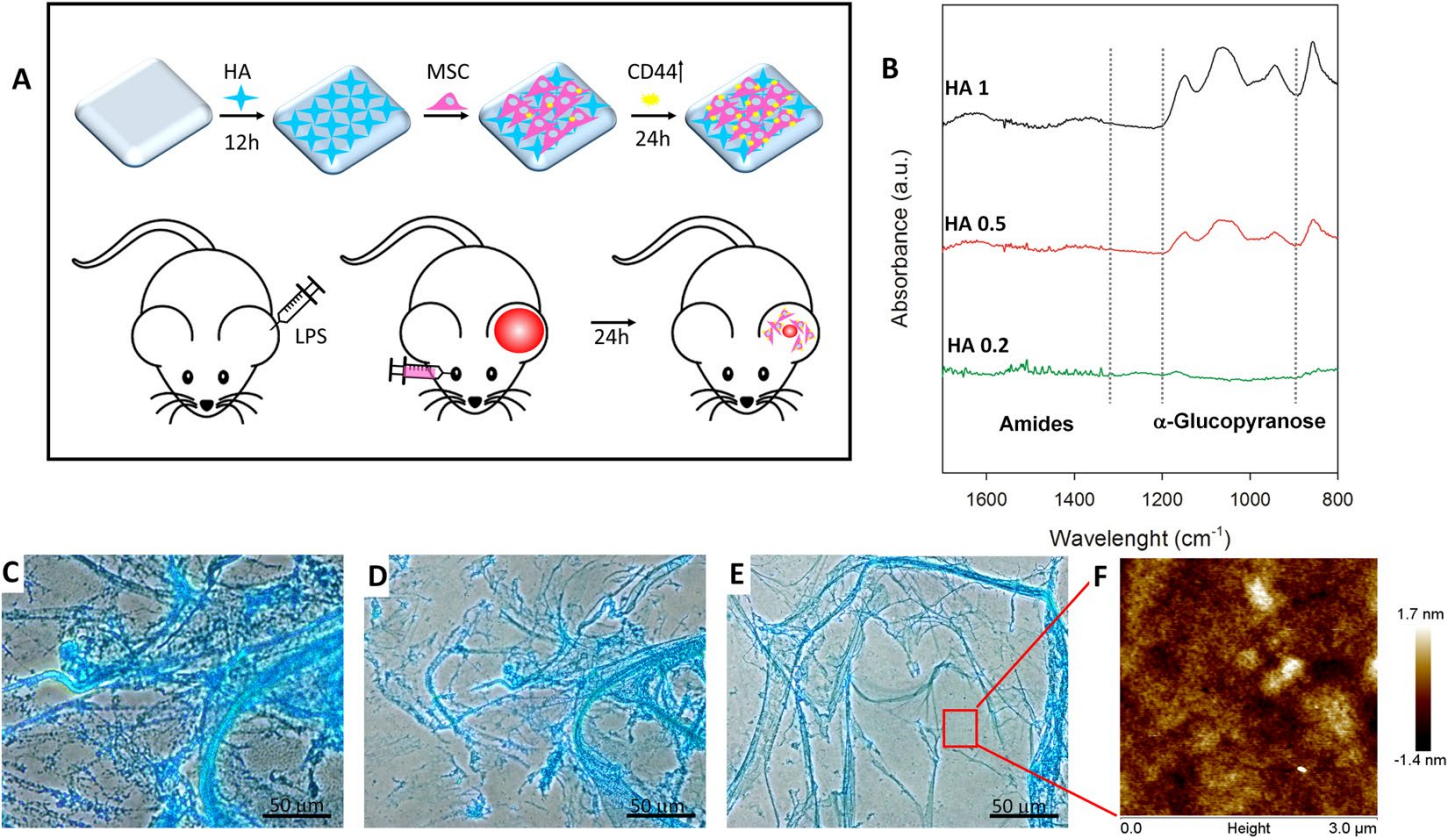
Schematic summarizing the proof of concept of the overall study (**A**). ATR-FTIR spectra of HA coated substrate at 0.2 (HA 0.2), 0.5 (HA 0.5), 1 (HA 1) mg/ml (**B**). Alcian Blue staining of the coating reveals the formation of a stable fibrotic coating on the surface of TCP (**C**) after 2 (**D**) and 4 washes (**E**). Atomic Force Microscope images of the surface show a thick and uniform layer of HA at the nanoscopic level (**F**). AFM- tip scratching showed the thickness of the HA multi-layers absorbed onto the TCP surface.

Considering the proposed “hit-and-run” mode of action for MSC upon transplantation as well as the interest in improving stem cell-therapy homing efficiency, we hypothesized that a simple approach would lead to a rapid targeting of MSC to inflammatory sites and facilitate localized secretion of potent biological factors. To test this hypothesis, we developed a system capable of promoting the transient expression of CD44 on the membranes of MSC, while leading to an improved homing affinity to the site of inflammation. Following the coating of tissue culture plates (TCP) with varying concentrations of HA (ranging from 1 mg/ml to 0.01 mg/ml), murine bone marrow-derived MSC were seeded onto HA coatings and CD44 expression was evaluated at the molecular and protein level at 1, 3 and 7 days. An *in vitro* migration assay was performed to determine whether the overexpression of CD44 could lead to enhanced homing potential and an *in vivo* lipopolysaccharide (LPS)-induced inflamed ear murine model was used to confirm whether HA-treated MSC displayed enhanced homing potential toward distant sites of inflammation following systemic administration. Lastly, pro- and anti-inflammatory genes and identification of anti-inflammatory macrophages were analyzed to assess the effectiveness of the system in supporting a predominant regenerative microenvironment. Figure 1A summarizes the idea of the whole study.

## Results

We investigated the TCP surface changes after HA treatment. The chemical structures of HA/TCP surfaces were first analyzed by ATR-FTIR spectroscopy. As shown in Fig. 1B, only HA 1, HA 0.5, and HA 0.2 coatings, showed the typical hyaluronic vibration modes: 1650–1510 (amides region), and 1050 cm−1 (C−O−C stretching vibrations of the α-glucopyranose structure), suggesting that only these coatings were thick enough to be detected on the TCP substrates.

To assess the glycosaminoglycan adsorption on the substrate in more detail, we performed Alcian Blue staining as described in the Methods section. Figure 1C represents the coating deposition at 1 mg/ml following two (Fig. 1D) and four (Fig. 1E) PBS washes. Microscopically HA coating is concentration dependent. Fibers covering surfaces were only found at the highest concentrations (1 mg/ml and 0.5 mg/ml) and disappeared at the lowest concentration. The nano-topography of the surface was also analyzed. Figure 1F shows the AFM pictures coated TCPs treated with HA1. Similarly, HA 0.5 and 1 revealed a compact surface coating, confirming prior observations. Mean roughness (Ra) of the uncoated TCPS was 2.05 ± 0.5 nm, increasing steadily to reach 10.05 ± 0.5 nm after HA1 treatment.

### Cell proliferation, morphology, and MSC-associated phenotype maintenance

**Figure 2:**
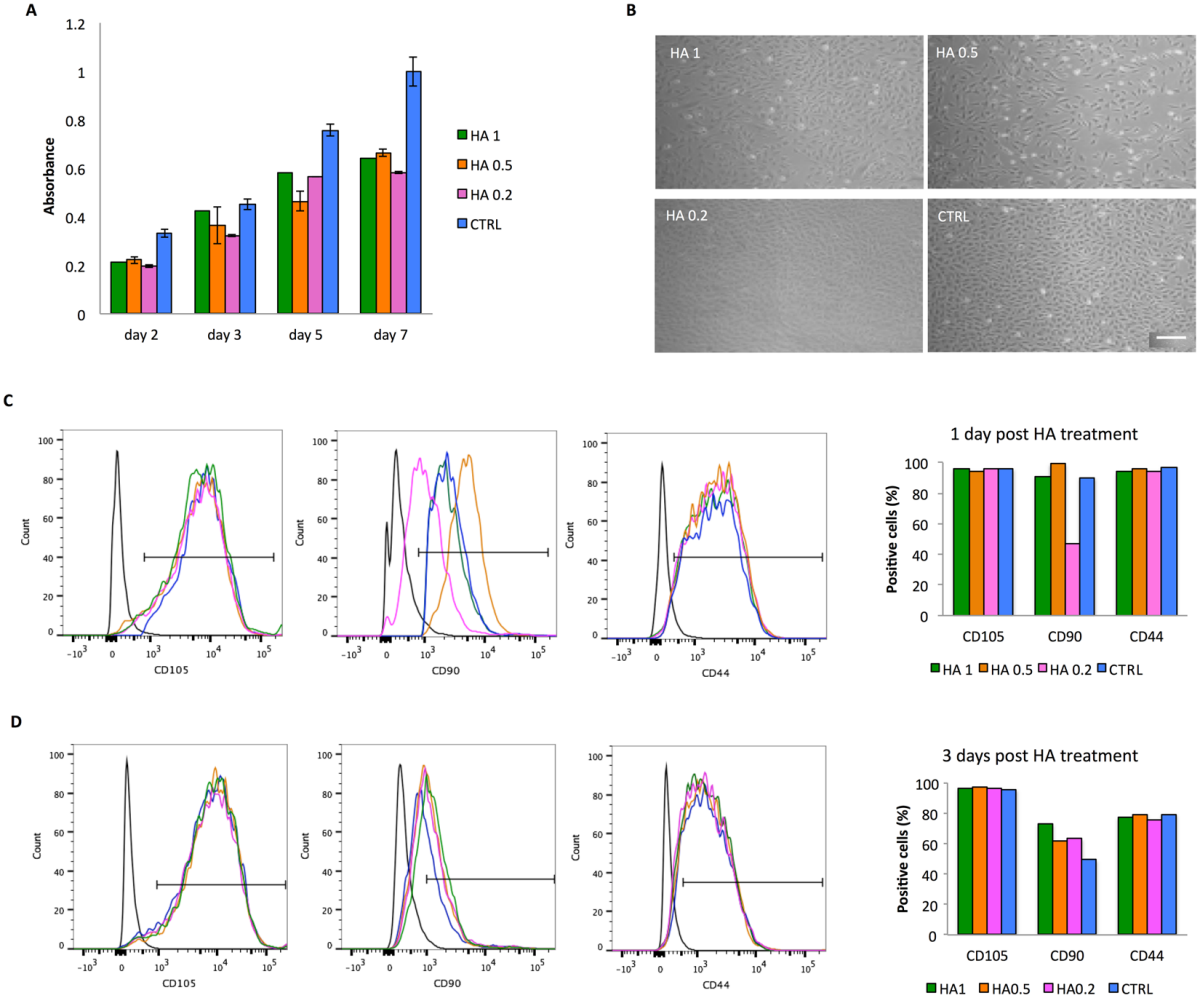
Effect of HA dilutions (0.2–1 mg/ml) on MSC viability, morphology, and surface markers. Temporal changes in the %AB are shown as representative of the presence of metabolically active cells over time revealed by Alamar blue assay **(A)**. A cumulative increase in %AB reduction reflects ongoing cell proliferation when exposed to serial HA dilutions (range). MSC grown in absence of HA are also reported for comparison (CTRL). Data are shown as mean of three independent biological replicates ± SD. Images representing cell morphology at day 1. 10X magnification. Scale bar: 20 μm (**B**). Histograms from flow cytometry analysis showing the percentage of cells positive for MSC-associated markers (CD105, CD90, and CD44) at 1 (**C**) and 3 (**D**) days post HA-treatment. MSC grown in absence of HA are reported for comparison (CTRL). Graphs showing the quantification of cells positive for the tested markers are reported on the right side.

**Figure 3:**
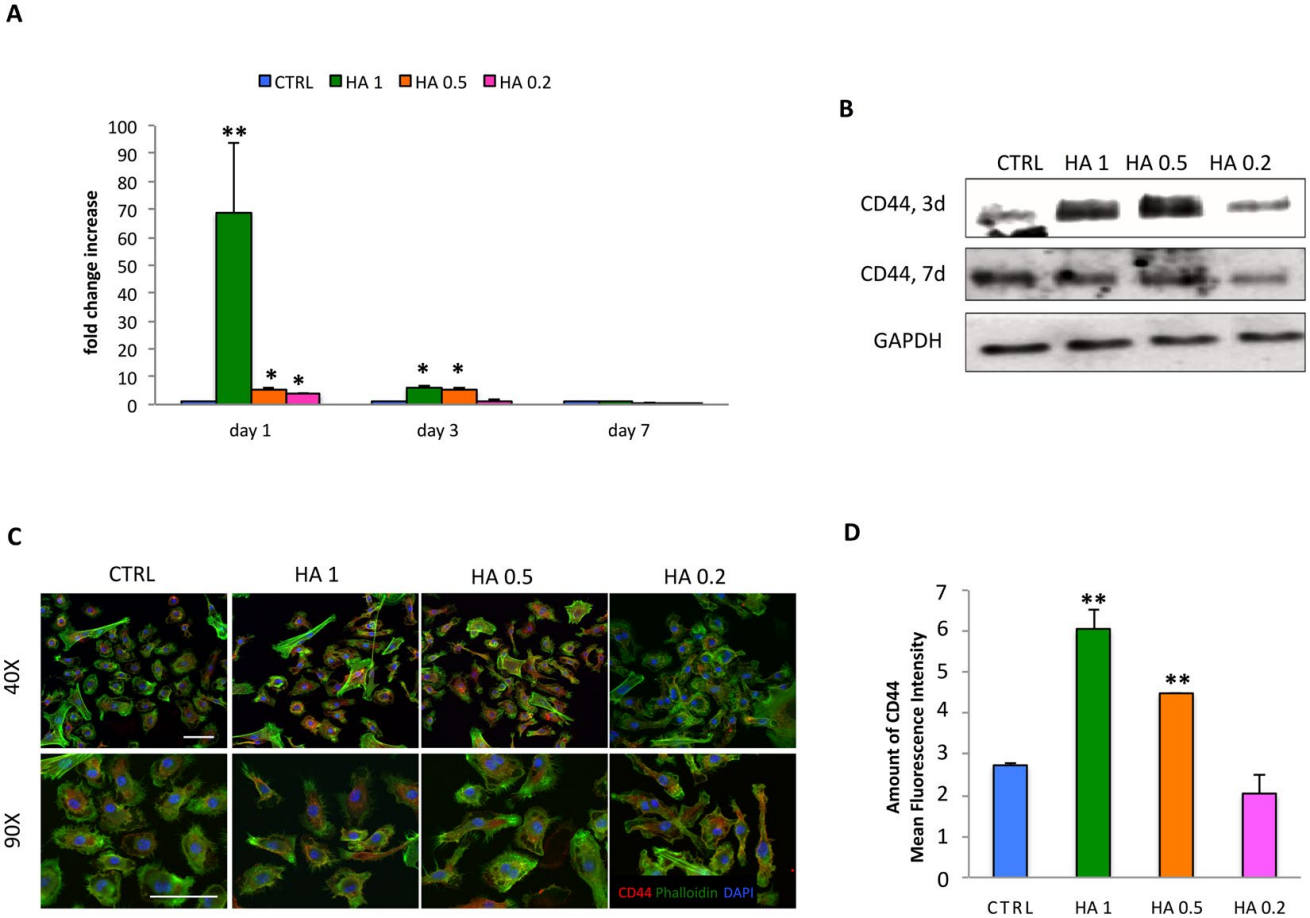
Effect of HA dilutions (0.2–1 mg/ml) on CD44 expression. Comparison between CD44 mRNA levels on MSC grown onto different concentrations HA (HA1, HA 0.5, and HA 0.2) for 1, 3 and 7 days (**A**). Data are represented as fold-change compared with the expression levels found in the untreated cells, CTRL (n = 3; * p < 0.05, **p < 0.01). Evaluation of CD44 expression on protein extracts from MSC treated with HA (HA 1, HA 0.5, and HA 0.2) or untreated (CTRL) after 3 and 7 days (3d and 7d, respectively). GAPDH was used as loading control (**B**). Immunofluorescence staining of MSC for CD44 (red), DAPI (blue) and Phalloidin (green) following 1-day exposure to HA at different concentrations. 40X and 90X magnifications. Scale bars: 50 μm (**C**). Quantification of CD44 mean fluorescence intensity on MSC. Asterisks depict significant (**p < 0.01) differences between cells exposed to HA (HA 1, HA 0.5, and HA 0.2) and untreated cells (CTRL) (**D**).

Alamar blue assay demonstrated that HA coatings support metabolically active cells growth during a period of 7 days (Fig. 2A) although HA-treated cells are generally slower in growth respect to controls. As demonstrated in Fig. 2B No changes in morphology are observed among different HA concentrations. Flow cytometry demonstrated HA-treated MSC did not affect the expression of the MSC-associated markers at 1 (Fig. 2C) and 3 (Fig. 2D) days compared to controls (CTRL). Minor fluctuations at 1 and 3 days in the positive expression of CD90 were observed following the treatment of MSC with HA 0.2.

### CD44 expression

After culturing MSC for 24 hrs onto TCP, a general increase in the levels of CD44 was found compared to control media when cells were exposed to HA. The greatest level of gene expression was assessed as a consequence of exposure to HA 1 and HA 0.5, with values assessed around 74.60 ± 5.5 and 12.60 ± 1.7, respectively (Fig. 3A). Such expression returned to the basal levels following exposure to HA, with the only exception of HA 1 and 0.5 (6.18 ± 0.42 and 5.43 ± 0.41), whose values decreased but were found higher than the control groups. In all cases, CD44 gene expression was comparable to controls after 7 days. Furthermore, a transient increase in CD44 expression following treatment with HA-coatings compared to controls was observed at the protein level (Fig. 3B). Higher levels of expression were detected by Western blot at day 3 and returned to baseline at day 7. At day 1, qualitatively (Fig. 3C) and quantitatively immunocytochemistry analysis (Fig. 3D) confirmed a significant increase in CD44 mean intensity/cell when cells were grown using TCP coated with the greatest concentration of HA (HA 1).

### *In vitro* migration assay

**Figure 4:**
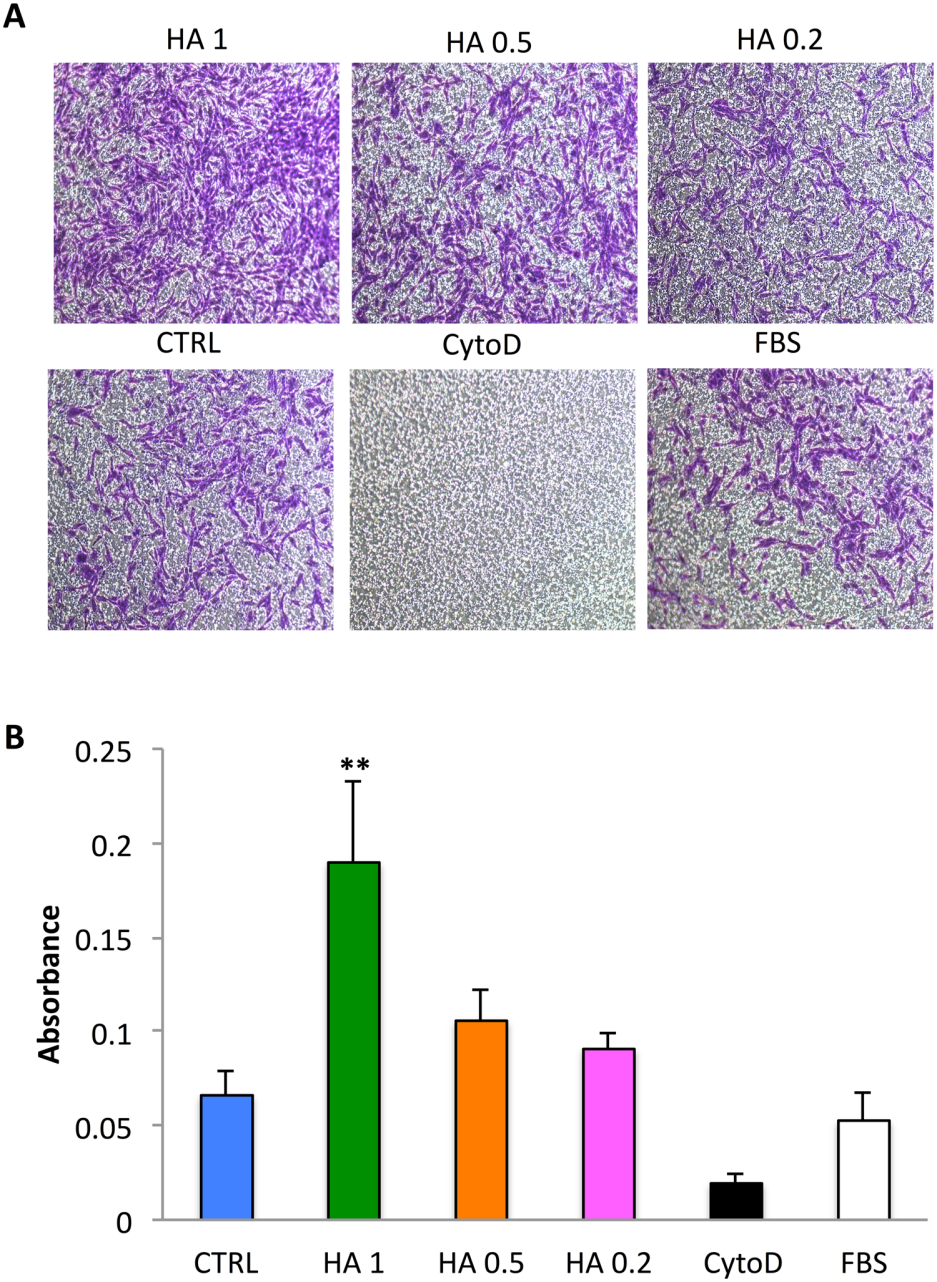
Homing potential of MSC exposed to HA (0.2–1 mg/ml). Following 24 hrs exposure to HA a transwell migration assay was performed. Migrated cells were stained with 0.2% crystal violet, imaged (**A)**. 20X Magnification. Scale bars: 20 μm. Quantification of migrated cells (**B**). Data are presented as the mean number of migrated cells ± SD. **P < 0.01 compared with the untreated cells (CTRL). Cells treated with FBS (10%) and cytochalacin D (CytoD) were used as controls.

An *in vitro* migration assay revealed both a qualitatively (Fig 4A) and quantitatively (Fig. 4B) improvement in cell migration following seeding on HA-coated TCP. Specifically, a 2.87-increase in the migratory potential of MSC was observed relative to untreated cells following exposure to HA 1.

### *In vivo* study

**Figure 5:**
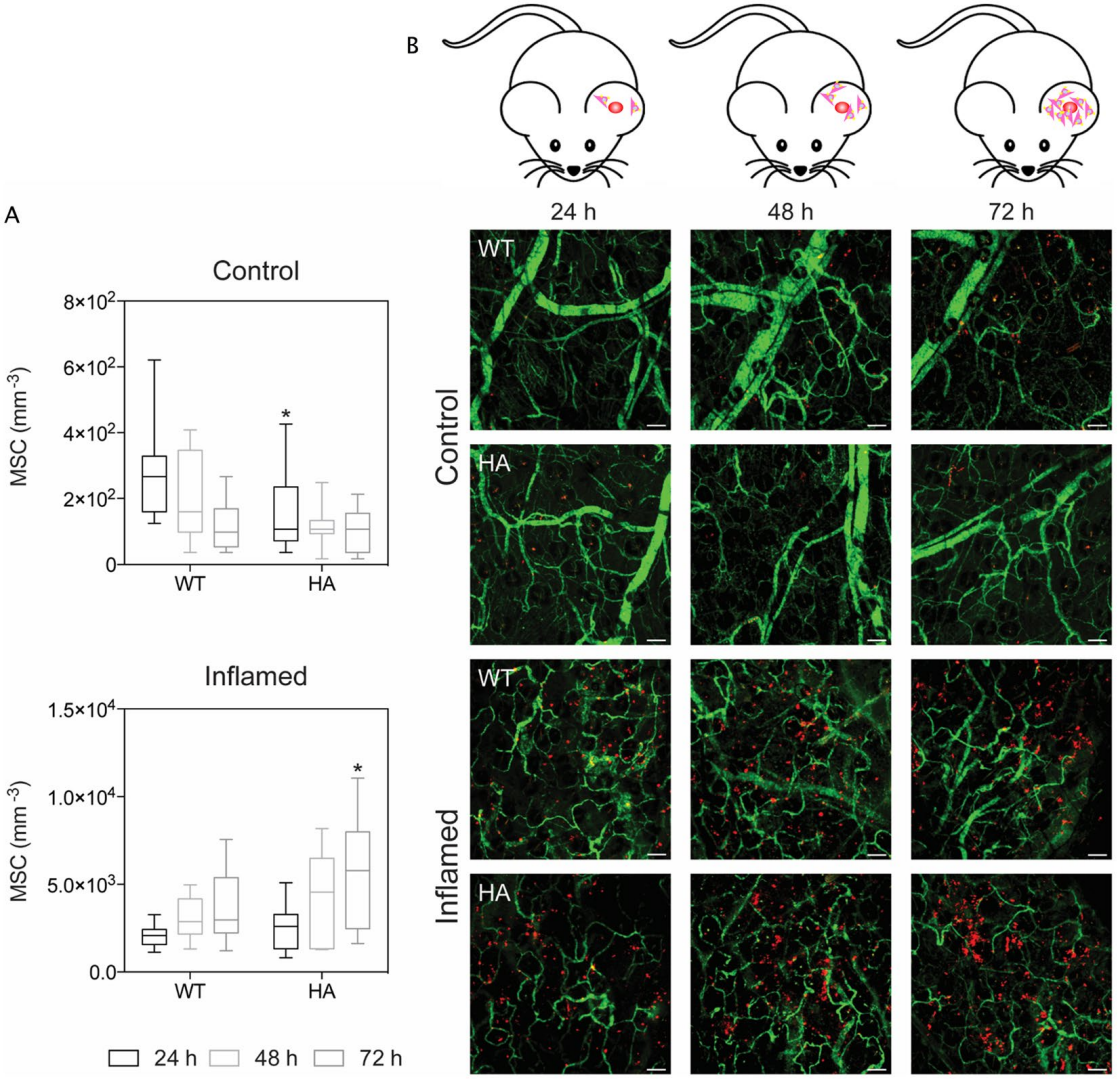
*In vivo* migration of MSC towards the inflammatory site. Representative maximum intensity projection images of control (top) and inflamed (bottom) ears at 24, 48, and 72 hrs after systemic administration of MSC (**A**). Vessels are shown in green and MSC in red. Scale bar: 100 µm. *p-value < 0.05. Quantification of MSC that successfully homed to control (top) or inflamed (bottom) ears **(B)**. Graphs are shown as box plots with the whiskers showing the minimum and maximum data points in each set.

**Figure 6:**
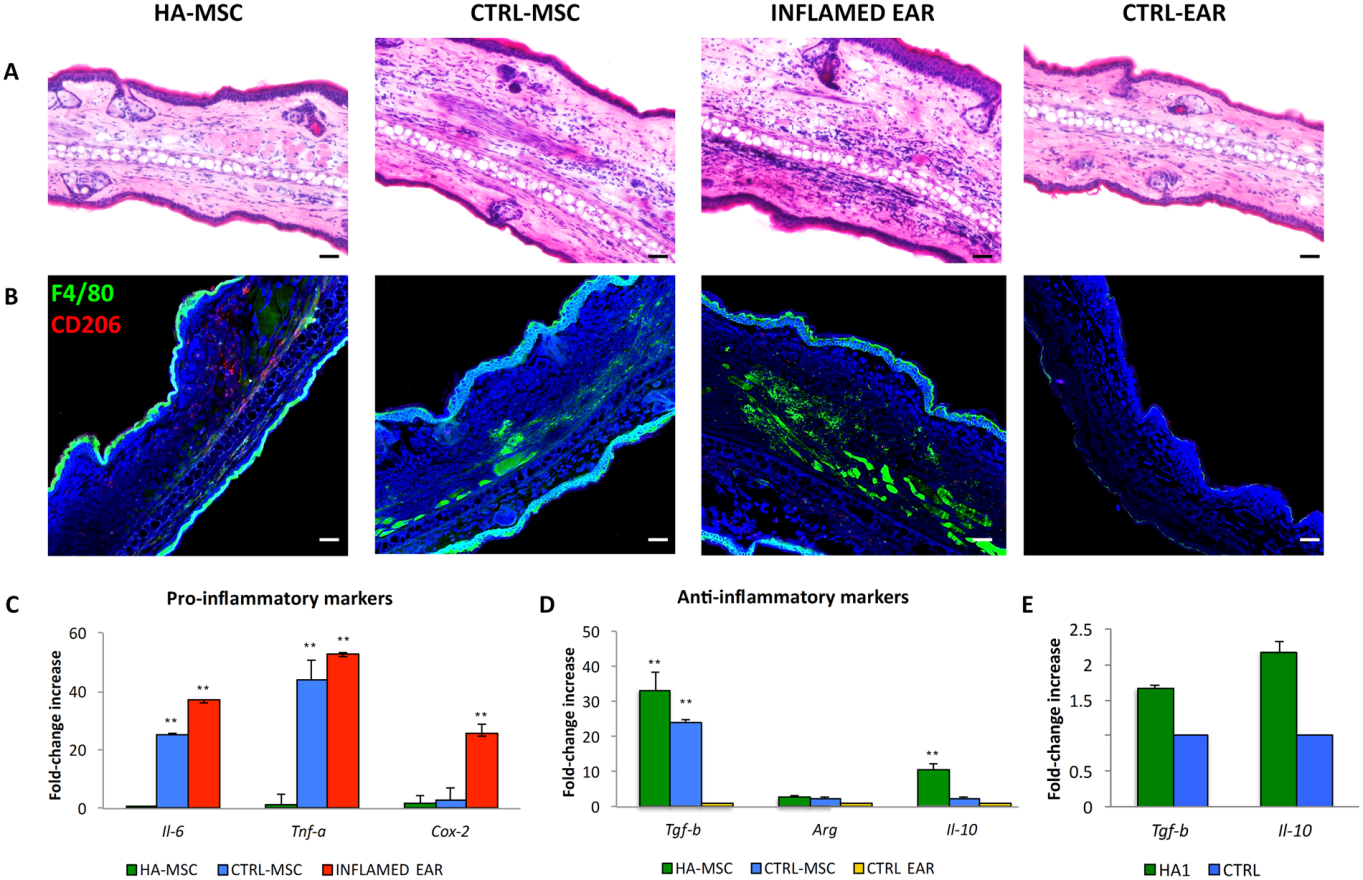
Reduction of inflammation following HA-treated MSC administration. Histological analysis of ear tissues (scale bars: 10 μm) (**A**). The sections shows an alteration of tissue architecture in the inflamed ear and in the ones treated with MSC. Immunofluorescence analysis of ear sections at 72 hrs reveals that HA-MSC exhibited a significant reduction in macrophages infiltration compared with the other groups. Scale bars: 20 μm (**B**). Moreover HA-MSC treatment enhanced the recruitment of anti-inflammatory macrophages (CD206^+^). Quantitative polymerase chain reaction for the expression of pro- (*Il-6, Tnf-a*, and *Cox-2*) and anti- (*Tgf-β, Arg, Il-10*) inflammatory genes (**C** and **D**, respectively) on explants 72 hrs following the injection of MSC grown onto HA-coated TCP (HA-MSC) and untreated MSC (CTRL-MSC). The expression levels of pro-inflammatory molecules found in the inflamed ears (INFLAMED EAR) are also shown for control ear with no inflammation (CTRL EAR), used as baseline (n = 3; *p < 0.5, **p < 0.01). Expression of *Tgf-β* and *Il-10* mRNA levels in MSC treated with HA1 (n = 3), before cell injection compared to MSC grown in 2D conditions (CTRL) **(E**).

The ability of HA to promote the homing of MSC to an inflamed site *in vivo* was evaluated by establishing a local inflammatory site in the ears of mice using LPS^29^ and imaging accumulation kinetics of MSC with IVM (Fig. 5A). Quantification and images at 24, 48, and 72 hrs revealed that both WT and HA-treated MSC selectively targeted inflamed ears to a greater extent than the contralateral healthy ear (Fig. 5B). Fluorescent images revealed a linear increase in cell count at each time point with a greater amount of cells found in inflamed ears. In addition, macroscopic observations of the ear showed gross signs of improvement in the mice treated with HA-MSC, in comparison with untreated MSC (Supplementary Figure 2A). Hematoxylin and eosin (H&E) staining confirmed this observation (Fig. 6A). H&E images showed normal ear tissue architecture for control group (left ear), whereas controls resulted in a substantial alteration of the ear’s architecture, increased neutrophil infiltration, and edema. However, immunofluorescence staining for anti-inflammatory macrophages (F4/80 + CD206 + , Fig. 6B) demonstrated higher infiltration of this population in the inflamed ears of mice treated with HA-MSC in comparison with all controls (Supplementary Figure 2B).

### *In vivo* molecular analysis

Concomitant with the retention of MSC at the site of inflammation, a reduced expression of pro-inflammatory genes (*Il-6* and *Tnf-a*) was observed when quantitative RT-PCR was performed on explanted ears 72 hrs after injection (Fig. 6C). Data obtained revealed that the expression of such genes dropped to baseline when cells were previously exposed to HA, whereas it was still comparable to that observed in the positive control represented by inflamed ear (25 ± 4.08 vs 37 ± 2.64, respectively). Concurrently, a statistically significant increase in the expression of anti-inflammatory genes (*Tgf-β* and *Il-10*) was observed in the tissue when cells had been previously treated with HA (Fig. 6D). The values obtained for the expression of *Tgf-β* were assessed around 33.13 ± 5.4 and 24.15 ± 0.80, respectively, compared to control ear. The levels of expression for *Il-10* were assessed 5-fold increase in HA-treated cells compared to control cells.

To exclude the possibility that the observed increase in *Tgf-β* and *Il-10* expression was induced by the HA treatment, the expression of these markers was evaluated before cells’ injection. As demonstrated in Fig. 6E, exposure to HA induced a slight increase in *Tgf-β* (1.65 ± 0.05 and 2.17 ± 0.14, respectively, compared to control (2D cells, value = 1).

## Discussion

MSC are immature cells able to self-renew and differentiate toward a plethora of cell types belonging to the three germinal layers. First used in regenerative medicine in the late 1950s^30^, they have demonstrated therapeutic effectiveness in the treatment of several disorders, including cardiovascular^31^, degenerative diseases affecting tendons^32^ lung^33^, liver^34^ and muscles^35^. Their regenerative feature is mainly due to their potential to home toward the site of injury (or inflammation) and to recreate a regenerative-permissive environment by releasing paracrine signals able to target endogenous cells^2, 36, 37^. Their use in human cell-based therapy is considered to be safe and, recently, we reported a complete list of the existing MSC clinical trials with no harmful effects over a 6.8-year period^1^. Following local or intravenous administration *in vivo*, MSC homing toward the site of injury/ inflammation relies upon their ability to interact with the cellular and chemical components of the microenvironment to ensure the correct anchorage and function. MSC mode of action has been reported not pass through their potential to engraft where required but in a “hit and run” manner^38^. As such, several groups have developed strategies to enhance therapeutic impact as well as their capability to migrate, localize and being retained at the site of injury, to improve their therapeutic efficacy^39^. Such approaches are mainly based on the modification of key molecules and ligands whose expression/activation can enhance MSC chemotaxis and adhesion and lead to an improved anti-inflammatory response^40^. Particular attention has been given to the role of the interaction between hyaluronan (HA) and its receptor on MSC membrane^41^. An increased stromal production of the HA is generally associated to inflammation^42^, which plays several functions, including the creation of a low resistance highly hydrated extracellular matrix thought to facilitate local cellular trafficking^43^.

Interestingly, the CD44-HA interaction is involved in the migration of exogenous MSC to damaged tissues. Deficits in receptor binding to selectins and/or their ligands (L-selectin, and their E-selectin ligand CD44) make MSC nonfunctional^44^, whereas through an enzymatic fucosylation procedure it is possible to increase CD44 epitope on MSC and activate a strong binding to endothelial E-selectin, resulting in effective MSC rolling on endothelial cells and the consequent extravasation into bone marrow sites^45, 46^. Other groups around the world have addressed the role of CD44 receptor and its ligand HA in the recruitment of injected MSC in a mouse model of glycerol-induced acute renal failure^27^ and in a rat chronic renal failure model^47^. In both cases, culture media was supplemented with soluble HA and proven to be sufficient in activating its receptor on the MSC surface and consequently improving the chemotactic potential, which was abolished with MSC pre-incubation with a blocking anti-CD44 monoclonal antibody.

With this in mind, we sought to propose a simple tool to exploit the HA-CD44 interaction and induce the transient overexpression of the hyaluronan receptor on the MSC membrane, which ultimately leads to an enhanced homing potential, both *in vitro* and *in vivo*. As such, we prepared HA-coated TCP with a serial HA dilution and tested the effect of each coating on MSC behavior. The analysis of the substrates by FTIR indicated that a relatively thick layer of HA was present on TCP surfaces, since extra thin layers (Å-size) would be very difficult to detect with this technique.

Although the coating presented a microscopic topography, this did not induced any differences in terms of cell morphology compared to the controls (untreated cells) at any time point (data not shown), suggesting no alteration in the molecular machinery activated in cells. Conversely, MSC grown onto HA-coatings showed a reduced proliferative potential with respect to the controls, typically indicative of the physiological need of cells to properly reorganize into a biomimetic material^48^. According to previously published studies, the exposure to HA induced a significant overexpression of CD44 as revealed at a molecular and protein level^49^ at 1 day in culture, especially for the highest concentration of HA employed (1 mg/ml). The transient nature of such treatment was supported by the noticeable decrease in CD44 expression at 3 days, which completely returned to baseline after 7 days. We then set up functional assays to correlate cell-surface receptor expression to *in vitro* and *in vivo* migration, and to a therapeutic response. The *in vitro* migration assay demonstrated that the 24-hrs treatment with the highest concentration (HA 1) was the most efficient in enhancing cell homing. Data obtained supported previously reported evidences, suggesting HA significantly promoted MSC migration^50, 51^, although discrepancies with literature were found regarding the most effective HA concentration. As an example, Bian *et al*. suggested the effect of CD44-HA on MSC to be dose-dependent, with the 200 μg/ml HA to be the most effective in improving MSC behavior and the concentrations of 100–300 μg/ml HA having weaker but similar ability^47^. We hypothesize that the differences observed are likely due to the binding of CD44-positive MSC to an HA substrate and structure (which is our case), or of a soluble HA molecule to the surface of a CD44-positive cell^52^. This feature has been reported to be common for interactions involving cell surface adhesion receptor-ligand interactions and is particularly relevant in the case of CD44 as it binds a highly repetitive ligand (i.e., HA)^53^. To evaluate whether our HA-coatings on TCP could increase CD44–HA interaction and provide a dominant force in guiding MSC to the inflamed site *in vivo*, we tested the potential of MSC grown on HA-coated TCP at the concentration of 1 mg/ml for 1 day in an ear model. Consistent with other studies^54^, HA-MSC were detectable as quickly as 24 hrs following injection, with a significant increase after 72 hrs. In addition, the recruitment and accumulation of MSC within the inflamed ear led to a decreased expression of pro-inflammatory markers, confirming previous works by Herrera HB *et al*.^27^ and demonstrating that the localization of exogenous MSC at the site of inflammation is dependent on the CD44–HA interaction. Immunohistochemistry analysis following systemic administration demonstrated that HA-treated MSC generated a strong anti-inflammatory effect *in vivo*. The combination of reduced neutrophil infiltration in comparison with inflamed and MSC controls, together with the early localization of M2 macrophages (F4/80^+^/CD206^+^) strongly suggest the high anti-inflammatory potential of the HA treatment. Furthermore, the increase expression of *Il-10* and Tgf-*β* levels in the inflamed ears following the treatment further validates these findings at a molecular level. Likely, the significant reduction of the local inflammation following systemic administration of HA-treated MSC was dependent on rapid localization of MSC to the inflamed site. Altogether our data confirm the role of CD44-HA interaction as a potential feature of MSC to be exploited to improve the therapeutic efficacy of MSC-based therapies. The system proposed herein is an easy, ready-to-use, and versatile tool to support the transient overexpression of CD44 on the MSC membrane, which plays a critical role in increasing systemically administered cell homing toward the site of inflammation.

## Methods

### Coating preparation and characterization

Tissue culture plates substrates (24 well plates) were coated with 1 ml of hyaluronic acid solutions (ACROS Organics™) of concentrations 0.1, 0.2, 0.5, and 1 mg/ml in PBS overnight at 4 °C (respectively called HA 0.1, HA 0.2, HA 0.5, HA 1). Uncoated wells were used as negative controls (CTRL). Before the use, the wells were washed with sterile PBS in order to remove the aggregates. In order to verify the presence of HA coating, Alcian blue 8 G (Sigma-Aldrich) was used to label hyaluronic acid (blue stain). Briefly, after coating the plate, 100 μl of Alcian Blue 1% in 3% acetic acid was added to the plate for 30 min and then washed with PBS. Images of plates’ surface were taken by Nikon inverted microscope and examined by Nikon Element.

The infrared spectra of TCP coated with different HA solution were obtained using a Fourier transform infrared (FTIR) using a Nicolet 6700 spectrometer (Thermo-Fisher Scientific, Waltham, MA). The samples were analyzed in attenuated total reflection (ATR) mode at 2 cm^−1^ resolution 256 times over the range of 500–2,000 cm^−1^ using the ATR/FTIR spectra following background subtraction, baseline correction, and binomial smoothing (11 points).

AFM images of the coated samples were collected in tapping™ mode by a Nanoscope V using single-beam silicon cantilever probes (RTESP: resonance frequency 300 KHz, nominal tip radius of curvature 10 nm, force constant 40 N/m). If necessary, data sets were subjected to a first-order flattening. Roughness (Ra), an arithmetic value that describes the absolute height of a surface in comparison to a two-dimensional plane represented by the average sample height was calculated using Nanoscope 6.13R1 software (Digital Instruments, NY, USA). Mean values from 4 random fields per sample in 3 independent experiments are reported. AFM tip scratch was performed on HA 0.1. A defect 1 um × 1um was introduced using AFM operating in contact mode with a Tap525 probe (Bruker, Sb (n) doped Si, nominal spring constant 200 N/m, nominal resonance frequency of 525 kHz). A set point of + 10 V (maximum applied force) was used to scan the selected area at 2 Hz.

### Tissue collection

BALB/c mice were sacrificed by carbon dioxide inhalation after isofluorane sedation in accordance with the regulatory guidelines of the Institutional Animal Care and Use Committee. A 70% (v/v) ethanol solution was applied liberally to the lower half of the animal to achieve a level of sterility prior to harvesting the tissues. Tibias and femurs were removed and cleaned rigorously with a sterile scalpel to remove excess muscle, tendons, periosteum, and connective tissues. The cleaned bones were kept on ice-cold PBS containing 2% (v/v) fetal bovine serum (FBS) for further processing.

### Cell isolation and culture

Mesenchymal stem cells were isolated from the bone marrow of healthy BALB/c as previously described^2, 55^. Briefly, bone marrow was flushed and plated into a large petri dish with alpha MEM supplemented with 20% fetal bovine serum (FBS, Thermo Scientific). For maintenance of cultures, cells were plated at up to 1 × 10^4^ cells/cm^2^ and incubated at 37 °C in a humidified atmosphere (90%) with 5% CO_2_, 5% O_2_. The number of viable cells was counted by the trypan blue dye exclusion method, using a Burker chamber. Adherent cells were serially passaged using TrypLE^TM^ Express (Invitrogen) upon reaching near confluence (80%) and then reseeded for culture maintenance. For *in vitro* and *in vivo* studies MSC were used at passage 3.

### MSC characterization before HA-treatment

Upon reaching confluence, cells were collected and characterized for the expression of MSC-associated markers (CD44, CD90, and CD105) and for the absence of the hematopoietic associated marker (CD45) with a FortessaTM cell analyzer (Beckton Dickinson), under the assistance of the HMRI Flow Cytometry Core. Antibodies used (PE anti-mouse/human CD44, APC/Cy7 anti-mouse CD45, PE/Cy7 anti-mouse CD105, and AlexaFluor 700 anti-mouse CD90) were purchased from BioLegend. Briefly, MSC were recovered using trypsin and spun down at 500 g for 5 min. They were then washed with FACS buffer labeled with directly conjugated antibodies according to manufacturer’s indications.

At passage 3 cells were also assessed for their capability to undergo osteogenic and adipogenic differentiation following a previously reported procedure^2^. Briefly, to induce osteogenesis MSC were seeded at the density of 5,000 cells/cm^2^ in 12-well plates. Osteogenic induction was performed over 14-day period using a StemPro Osteogenesis Differentiation Kit (Gibco). To confirm differentiation, conventional von Kossa and alkaline phosphatase staining were performed (VECTOR Blue Alkaline Phosphatase Substrate Kit; Vector Labs). For adipogenesis, cells were seeded at the density of 10^4^ cells/cm^2^. Induction was performed using the StemPro Adipogenic Differentiation Kit (Gibco) for a 21-day period. Intracellular lipid droplet accumulation was visualized by conventional Oil red O staining.

### Effect of HA treatment on cell viability, morphology, and surface markers expression

After ensuring the protocol used allowed for the isolation of a homogeneous MSC population positive for MSC-associated markers and negative for hematopoietic markers (Supplementary Figure 1A) and confirming its differentiative potential towards two mesodermal lineages (Supplementary Figure 1B), the effect of HA on their viability, morphology and phenotype was assessed. Proliferation of cells grown onto HA-coated TCP at concentrations ranging from 1 to 0.2 mg/ml was evaluated by Alamar Blue assay (Invitrogen, ThermoFisher Scientific) according to manufacturer’s instructions during a 7-day period. Cells were seeded onto HA-coated plates at the concentration of 5,000 cells/cm^2^. Optical density was measured at wavelengths of 570 and 600 nm. Because the culture medium was not changed during this period, the calculated percentage of Alamar blue reduction (%AB) is a cumulative value. Data are shown as mean of 3 independent biological replicates. Values are reported as %AB over time, which is associated with the presence of metabolically active cells. Data obtained from MSC grown in standard conditions are used for comparison. Cells were also monitored and imaged for morphology at the same time points. Fluctuation in the MSC-associated surface markers following the HA-treatment was assessed at different time points (1 and 3 days) by flow cytometry as reported above.

### Hyaluronic acid receptor (CD44) expression

#### Gene expression

Quantitative RT-PCR analysis was performed to evaluate *CD44* expression following HA treatment at 1, 3 and 7 days *in vitro*. Total RNA was isolated from HA-treated cells and explanted tissues using TRIzol reagent (Invitrogen). DNAse (Sigma) treatment followed the reaction. RNA concentration and purity were measured using a NanoDrop ND1000 spectrophotometer (NanoDrop Technologies). The cDNA was synthesized from 1 μg total RNA, using the iScript retrotranscription kit (Bio-Rad Laboratories), and quantitative PCR was run in the ABI 7500 Fast Sequence Detection System (Applied Biosystems) using commercially available master mix. The following target probe (Applied Biosystems) was used to evaluate the expression of *CD44*: Mm01277161_m1. Gene expression was normalized to the level of glyceraldehyde 3-phosphate dehydrogenase (*Gapdh*; *Mm99999915*_*g1*).

#### Western blotting assay

For the analysis of CD44 expression at a protein level following the treatment with HA at different concentrations (HA 1, HA 0.5, and HA 0.2) for 3 and 7 days, 1 × 10^6^ MSC were lysed using RIPA buffer (Thermo) mixed with halt protease inhibitor cocktail (Thermo). Untreated cells acted as control. Protein concentration was determined by Bradford assay (Bio-Rad) using BSA at known concentration (1 µg/µl) to build the standard curve. Forty μg of whole cell extract were loaded onto 12% acrylamide SDS-PAGE gel (Bio-Rad) and run for 40 minutes at 25 mA constant. Proteins were then transferred to a nitrocellulose membrane for Western blot analysis. Blots were incubated with primary anti-CD44 antibody (Abcam, 1:2000) and anti-GAPDH (Abcam, 1:20,000) overnight. Then, horseradish peroxidase (HRP)-conjugated secondary antibodies were used to incubate the membrane for 1 hr. Bands were visualized using a SuperSignal West Dura Chemiluminescent Substrate (Thermo) and images were acquired with ChemiDoc XRS + System and Image Lab software (Bio-Rad). Densitometry analysis was performed using ImageJ software (NIH).

#### Immunocytochemistry

After 1 day from exposure, cells were fixed with 4% formaldehyde in phosphate-buffered and stained for actin cytoskeleton with FITC-Phalloidin (Life Technology) and for CD44 with Alexa Fluor® 647 anti-mouse/human CD44 Antibody (Biolegend). After washing in PBS, samples were stained with anti-fading 4’,6-diamidino-2-phenylindole (DAPI; Life Technology) for 1 minute and captured on a confocal laser microscope (A1 Nikon Confocal Microscope). Images were acquired and analyzed with NIS-Element Nikon.

### Migration assay *in vitro*

Cell Migration Kit (including 24 well migration plate with well inserts, cotton tips, cell stain solution, and extraction solution) was purchased from Cell Solutions, Inc. HA-treated MSC (HA 1, HA 0.5, and HA 0.2) were seeded on the well inserts (1 × 10^5^ cells/insert) and incubated for 24 hrs. Untreated cells were used as positive control. Negative controls were represented by cells kept in FBS-free media with the addition of cytochalacin D (2.5 μg/μl) to inhibit MSC migration. Migration was calculated 24 hrs after cell seeding. Media was then aspirated from the well inserts and the interior of each insert was swabbed with wet cotton tips to remove any non-migratory cells. The inserts were then kept in 400 μl of cell stain solution, incubated for 10 minutes, washed with sterile water, and allowed to air dry. Cells were imaged before 300 μl of extraction solution was added to the bottom well and incubated for 10 minutes on an orbital shaker. 100 μl of each sample from the bottom well was transferred to a 96-well plate to measure absorbance on the spectrophotometer.

### Inflammation ear *in vivo* model

BALB/c mice ears (n = 17) were removed of hair using a depilatory cream (Nair®) and inflamed using *E. coli* LPS (Sigma Aldrich) in the right ear as previously described^56^. Animal studies were conducted following approved protocols (AUP-0515–0031) established by Houston Methodist Research Institute’s Institutional Animal Care and Use Committee (IACUC) in accordance with the guidelines of the Animal Welfare Act and the Guide for the Care and Use of Laboratory Animals. Briefly, mice were sedated and administered a single injection of 30 µg of LPS in PBS (1 mg/mL) into the base of the right ear. Similarly, 30 µL of PBS was injected into the base of the left ear. Before injection, MSC were plated onto HA 1-coated plates or in standard conditions and cultured for 24 hrs. Untreated and HA-treated MSC were stained using 10 μM DiD lipophilic tracer following manufacturer suggestions (Invitrogen) and filtered with a 40 µm cell strainer (BD). Next, a one-time, retro-orbital injection of 6 × 10^5^ MSC per mouse (i.e., untreated and HA-treated) was administered. The mice were then imaged 24, 48, and 72 hrs following MSC injection using a Nikon A1 intravital microscope. Just prior to imaging, 40 µL of FITC-dextran (70 kDa; Sigma Aldrich) was injected retro-orbitally for delineation of vasculature. Images were then acquired using a 50-µm z-stack with a step size of 5 µm and analyzed for the MSC present in each section using NIS Elements (Nikon).

### Histology and Immunofluorescence analysis

Explanted mice ears where cut by a biopsy pouch centered in the middle zone (diameter 8 mm), washed twice with PBS, embedded at the hedge in O.C.T. (Tissue-Tek O.C.T. Compound, Sakura Finetek), and instantly frozen in liquid nitrogen. 8-μm thick slides were obtained cutting ears block with a cryostat at −20 °C. For H&E staining, slides were thawed, hydrated, washed and stained with hematoxylin and eosin (Sigma-Aldrich). Immunofluorescence staining has been performed on consecutive ear sections. Briefly, slides were thawed and blocked with goat serum 5% (Sigma-Aldrich) PBS-T 1 × solution. After washing, they were incubated overnight at 4° with anti-macrophage antibody and anti- CD206 (Alexa Fluor 488 anti-mouse F4/80 and Brilliant Violet 605 anti-mouse CD206 Biolegend). Excess of the anti-neutrophil antibody was washed out with PBS 1X. Cells nuclei were stained with 4,6-diamidino-2-phenylindole (DAPI). Slides were sealed with ProLong Gold antifade reagent (Life Technologies). Images were captured with a Nikon Eclipse Ti Inverted Fluorescent Microscope.

### Inflammatory genes expression

The final outcome of the system, by means the reduction in ear inflammation mediated *in vivo* by HA-treated MSC (HA-MSC) compared to untreated MSC (CTRL MSC) was also evaluated on the explants. The following target probes (Applied Biosystems) were used to evaluate the expression of the following markers. Pro-inflammatory markers include *TNF-α* (Μm00443258_μ1), *IL-6* (Mm00446190_m1), and *Cox2* (Mm03294838_g1). Anti-inflammatory markers include *Tgf-β* (Mm00441727_g1), arginase (Mm00475988_m1) and *Il-10* (Mm01288386_m1). The mRNA levels of *Tgf-β* and *Il-10* were also assessed in HA-treated MSC before injection to exclude the possibility that the changes observed in anti-inflammatory markers expression were induced by the treatment itself. Gene expression was determined by qPCR following the procedure reported above.

### Statistical analysis

Statistical analysis was performed using GraphPad Instat 3.00 for Windows (GraphPad Software). Three replicates for each experiment were performed and the results are reported as mean ± standard deviation. *p *≤* 0.05* was considered as significant, *p < 0.01* highly significant. One-way ANOVA for multiple comparisons by Student-Newman-Keuls multiple comparison test was used.

## Electronic supplementary material

Supplementary Information

## References

[1] Corradetti B, Ferrari M (2016). Nanotechnology for mesenchymal stem cell therapies. J Control Release.

[2] Corradetti B (2015). Osteoprogenitor cells from bone marrow and cortical bone: understanding how the environment affects their fate. Stem Cells Dev.

[3] Lange-Consiglio A (2012). Characterization and potential applications of progenitor-like cells isolated from horse amniotic membrane. J Tissue Eng Regen Med.

[4] Lange-Consiglio A (2016). Equine Amniotic Microvesicles and Their Anti-Inflammatory Potential in a Tenocyte Model In Vitro. Stem Cells Dev.

[5] Lange-Consiglio A (2013). Investigating the efficacy of amnion-derived compared with bone marrow-derived mesenchymal stromal cells in equine tendon and ligament injuries. Cytotherapy.

[6] Muschler GF, Nakamoto C, Griffith LG (2004). Engineering principles of clinical cell-based tissue engineering. J Bone Joint Surg Am.

[7] Karp JM, Leng Teo GS (2009). Mesenchymal stem cell homing: the devil is in the details. Cell Stem Cell.

[8] Levy O (2013). mRNA-engineered mesenchymal stem cells for targeted delivery of interleukin-10 to sites of inflammation. Blood.

[9] Lee RH (2009). Intravenous hMSCs improve myocardial infarction in mice because cells embolized in lung are activated to secrete the anti-inflammatory protein TSG-6. Cell Stem Cell.

[10] von Bahr L (2012). Analysis of tissues following mesenchymal stromal cell therapy in humans indicates limited long-term engraftment and no ectopic tissue formation. Stem Cells.

[11] Ranganath SH, Levy O, Inamdar MS, Karp JM (2012). Harnessing the mesenchymal stem cell secretome for the treatment of cardiovascular disease. Cell Stem Cell.

[12] Wu Y, Zhao RC (2012). The role of chemokines in mesenchymal stem cell homing to myocardium. Stem Cell Rev.

[13] Hocking AM (2015). The Role of Chemokines in Mesenchymal Stem Cell Homing to Wounds. Adv Wound Care (New Rochelle).

[14] De Becker A, Riet IV (2016). Homing and migration of mesenchymal stromal cells: How to improve the efficacy of cell therapy?. World J Stem Cells.

[15] Naderi-Meshkin H, Bahrami AR, Bidkhori HR, Mirahmadi M, Ahmadiankia N (2015). Strategies to improve homing of mesenchymal stem cells for greater efficacy in stem cell therapy. Cell Biol Int.

[16] Huang J (2010). Genetic modification of mesenchymal stem cells overexpressing CCR1 increases cell viability, migration, engraftment, and capillary density in the injured myocardium. Circ Res.

[17] Cheng Z (2008). Targeted migration of mesenchymal stem cells modified with CXCR4 gene to infarcted myocardium improves cardiac performance. Mol Ther.

[18] Sarkar D (2011). Engineered cell homing. Blood.

[19] Brenner S (2004). CXCR4-transgene expression significantly improves marrow engraftment of cultured hematopoietic stem cells. Stem Cells.

[20] Kumar S, Ponnazhagan S (2007). Bone homing of mesenchymal stem cells by ectopic alpha 4 integrin expression. FASEB J.

[21] Dykstra B (2016). Glycoengineering of E-Selectin Ligands by Intracellular versus Extracellular Fucosylation Differentially Affects Osteotropism of Human Mesenchymal Stem Cells. Stem Cells.

[22] Merzaban JS (2011). Analysis of glycoprotein E-selectin ligands on human and mouse marrow cells enriched for hematopoietic stem/progenitor cells. Blood.

[23] Levy O (2015). A small-molecule screen for enhanced homing of systemically infused cells. Cell Rep.

[24] Sarkar D (2010). Engineered mesenchymal stem cells with self-assembled vesicles for systemic cell targeting. Biomaterials.

[25] Nusgens BV (2010). [Hyaluronic acid and extracellular matrix: a primitive molecule?]. Ann Dermatol Venereol.

[26] Solis MA (2012). Hyaluronan regulates cell behavior: a potential niche matrix for stem cells. Biochem Res Int.

[27] Herrera MB (2007). Exogenous mesenchymal stem cells localize to the kidney by means of CD44 following acute tubular injury. Kidney Int.

[28] Sackstein R (2008). Ex vivo glycan engineering of CD44 programs human multipotent mesenchymal stromal cell trafficking to bone. Nat Med.

[29] Molinaro, R. *et al*. Biomimetic proteolipid vesicles for targeting inflamed tissues. *Nature materials* (2016). 10.1038/nmat4644 PMC5127392 27213956

[30] Mathe, G., Jammet, H. & Pendic, B. Transfusions and grafts of homologous bone marrow in humans accidentally irradiated to high doses (1959). 13646287

[31] Psaltis PJ, Zannettino AC, Worthley SG, Gronthos S (2008). Concise review: mesenchymal stromal cells: potential for cardiovascular repair. Stem Cells.

[32] Awad HA (1999). Autologous mesenchymal stem cell-mediated repair of tendon. Tissue engineering.

[33] Ren C (2008). Therapeutic Potential of Mesenchymal Stem Cells Producing Interferon‐α in a Mouse Melanoma Lung Metastasis Model. Stem Cells.

[34] Kuo, T. K. *et al*. Stem cell therapy for liver disease: parameters governing the success of using bone marrow mesenchymal stem cells. *Gastroenterology* **134**, 2111–2121. e2113 (2008). 10.1053/j.gastro.2008.03.015 PMC3086672 18455168

[35] Pittenger M, Vanguri P, Simonetti D, Young R (2002). Adult mesenchymal stem cells: potential for muscle and tendon regeneration and use in gene therapy. Journal of Musculoskeletal and Neuronal Interactions.

[36] Anthony DF, Shiels PG (2013). Exploiting paracrine mechanisms of tissue regeneration to repair damaged organs. Transplantation research.

[37] Corradetti B (2014). Amniotic membrane-derived mesenchymal cells and their conditioned media: potential candidates for uterine regenerative therapy in the horse. PLoS One.

[38] Ankrum JA, Ong JF, Karp JM (2014). Mesenchymal stem cells: immune evasive, not immune privileged. Nature biotechnology.

[39] da Silva Meirelles L, Fontes AM, Covas DT, Caplan AI (2009). Mechanisms involved in the therapeutic properties of mesenchymal stem cells. Cytokine & growth factor reviews.

[40] Multon, M.-C., Rothblatt, J., Deleuze, J.-F., Lin, C. P. & Karp, J. M. A Small-Molecule Screen for Enhanced Homing of Systemically Infused Cells. 10.1016/j.celrep.2015.01.057 PMC4361231 25732817

[41] Qian H, Le Blanc K, Sigvardsson M (2012). Primary mesenchymal stem and progenitor cells from bone marrow lack expression of CD44 protein. Journal of Biological Chemistry.

[42] Sironen R (2011). Hyaluronan in human malignancies. Experimental cell research.

[43] Lu P, Weaver VM, Werb Z (2012). The extracellular matrix: a dynamic niche in cancer progression. The Journal of cell biology.

[44] Sackstein R (2008). Ex vivo glycan engineering of CD44 programs human multipotent mesenchymal stromal cell trafficking to bone. Nature medicine.

[45] Rüster B (2006). Mesenchymal stem cells display coordinated rolling and adhesion behavior on endothelial cells. Blood.

[46] Thankamony SP, Sackstein R (2011). Enforced hematopoietic cell E-and L-selectin ligand (HCELL) expression primes transendothelial migration of human mesenchymal stem cells. Proceedings of the National Academy of Sciences.

[47] Bian X-H (2014). The role of CD44-hyaluronic acid interaction in exogenous mesenchymal stem cells homing to rat remnant kidney. Kidney and Blood Pressure Research.

[48] Corradetti B (2016). Chondroitin Sulfate Immobilized on a Biomimetic Scaffold Modulates Inflammation While Driving Chondrogenesis. Stem cells translational medicine.

[49] Kobayashi H (2002). CD44 stimulation by fragmented hyaluronic acid induces upregulation of urokinase‐type plasminogen activator and its receptor and subsequently facilitates invasion of human chondrosarcoma cells. International journal of cancer.

[50] Zhu H (2006). The role of the hyaluronan receptor CD44 in mesenchymal stem cell migration in the extracellular matrix. Stem cells.

[51] Herrera M (2007). Exogenous mesenchymal stem cells localize to the kidney by means of CD44 following acute tubular injury. Kidney international.

[52] Lesley J, Hascall VC, Tammi M, Hyman R (2000). Hyaluronan binding by cell surface CD44. Journal of Biological Chemistry.

[53] Dustin ML, Springer TA (1991). Role of lymphocyte adhesion receptors in transient interactions and cell locomotion. Annual review of immunology.

[54] Kurtz A (2008). Mesenchymal stem cell delivery routes and fate. International journal of stem cells.

[55] Soleimani M, Nadri S (2009). A protocol for isolation and culture of mesenchymal stem cells from mouse bone marrow. Nat Protoc.

[56] Gaudet JM, Ribot EJ, Chen Y, Gilbert KM, Foster PJ (2015). Tracking the fate of stem cell implants with fluorine-19 MRI. PloS one.

