# Supplementary material for: Hyaluronic acid coatings as a simple and efficient approach to improve MSC homing toward the site of inflammation
Source: Sci Rep. 2017 Aug 11;7:7991. doi: 10.1038/s41598-017-08687-3 (PMC5554184; doi:10.1038/s41598-017-08687-3)
Supplement: Supplementary file 1 — Supplementary Information [file 41598_2017_8687_MOESM1_ESM.pdf]

Hyaluronic acid coatings as a simple and efficient approach to improve MSC homing toward the site of inflammation.

Bruna Corradetti[a,b#], *Francesca Taraballi*[c#], *Jonathan O. Martinez*[c], *Silvia Minardi*[c], *Nupur Basu*[c], *Guillermo Bauza*[c,d], *Michael Evangelopoulos*[c], *Sebastian Powell*[c], *Claudia Corbo*[c], *Ennio Tasciotti*[c,d*]

[a] Department of Nanomedicine, Houston Methodist Research Institute, Houston, TX 77030, USA; [b] Department of Life and Environmental Sciences, Università Politecnica delle Marche, 60131, Ancona, Italy; [c] Center for Biomimetic Medicine, Houston Methodist Research Institute, Houston, TX, 77030, USA: [d]Centre for NanoHealth, Swansea University Medical School, Swansea University Bay, Singleton Park, SA2 8PP, Wales, UK. [e]Department of Orthopaedic & Sports Medicine, The Houston Methodist Hospital, Houston, TX 77030, USA.

# Equal contribution

* Corresponding author

Email: etasciotti@houstonmethodist.org

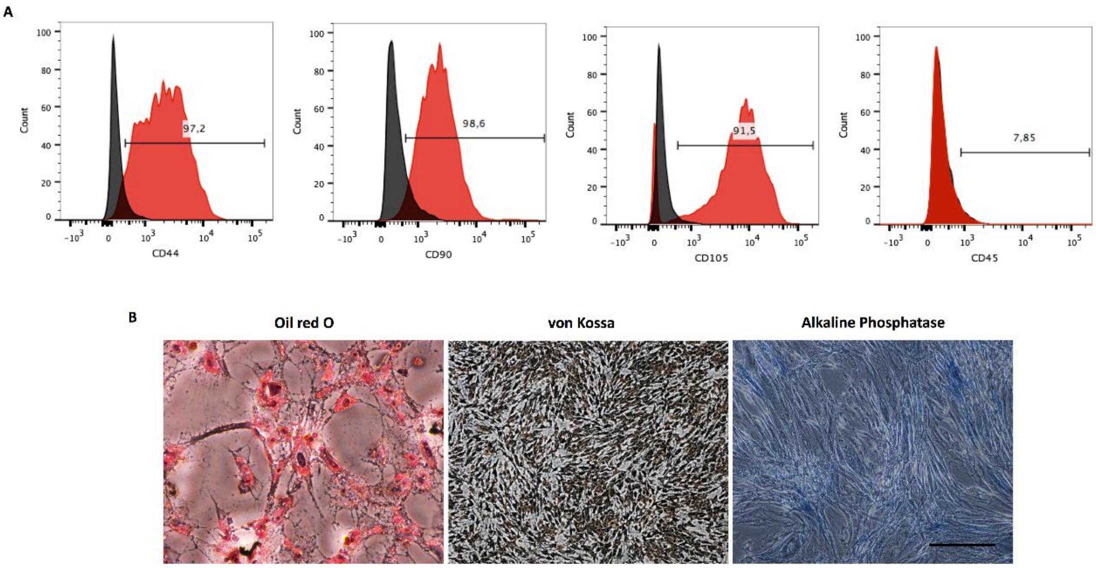

Supplementary Figure 1. MSC characterization. Flow cytometry analysis of P3 bone marrow-derived MSC demonstrating their positive expression for CD44, CD90, and CD105 and negative for the hematopoietic marker CD45 **(A)**. Adipogenic (Oil red O, 20x Magnification. Scale bar: 10 μm) and osteogenic (von Kossa and alkaline phosphatase, 10x magnification) differentiation of MSC at P3 **(B)**.

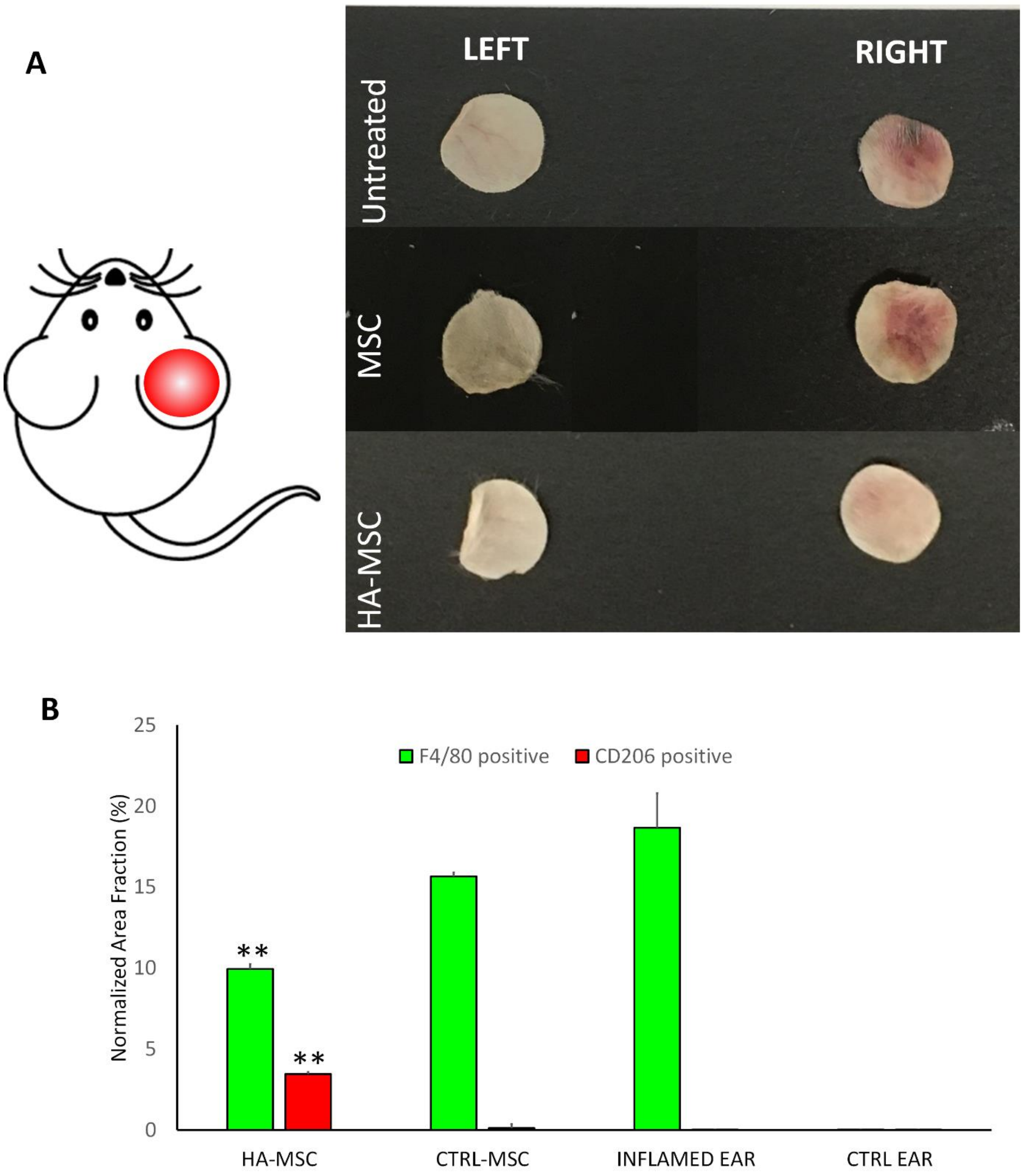

Supplementary Figure 2. Gross images of inflamed ears (**A**). Representative left (control) and right (treated) ears of mice (n=3) were harvested and punched (diameter 8 mm). Macroscopic observation revealed the classical signs of inflammation, redness and swelling. Quantification of F4/80 (macrophages) and CD206 (mannose receptor) marker expression in immunofluorescence

images (**B)**. Four sections per animal were stained and analyzed by NIS elements. (***p*<0.01).
